# Supplementary material for: Ivermectin exposition during neurulation induces neural tube defects and neuromuscular alterations in Xenopus laevis through purinergic P2X4-signaling
Source: Front Pharmacol. 2026 Aug 24;17:1870145. doi: 10.3389/fphar.2026.1870145 (PMC13546998; doi:10.3389/fphar.2026.1870145)
Supplement: Supplementary file 1 [file DataSheet1.pdf]

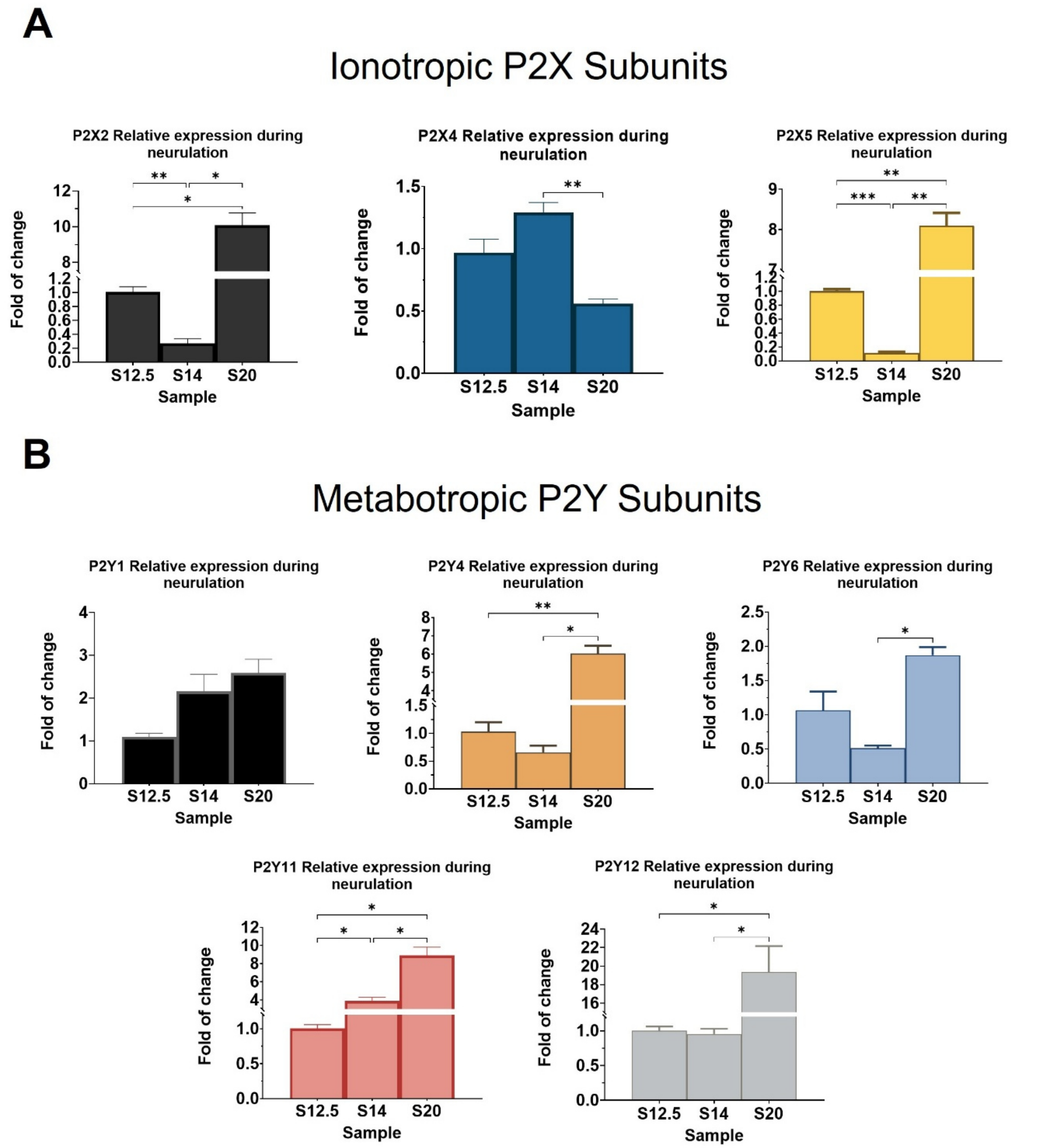

Supplementary figure 1. **RT-qPCR screening analysis of purinergic subunits during neurulation.** (A) Fold of change of ionotropic P2X2,4,5 mRNA subunits normalized to stage 12.5. (B) Fold of change of metabotropic P2Y1,4,6,11,12 mRNA subunits normalized to stage S12.5. N=3. Brown-Forsythe and Welch ANOVA, Dunnet's correction, (* $P<0.05$, ** $P<0.01$, *** $P<0.001$). The error bars of the data correspond to the standard error of the mean (SEM)

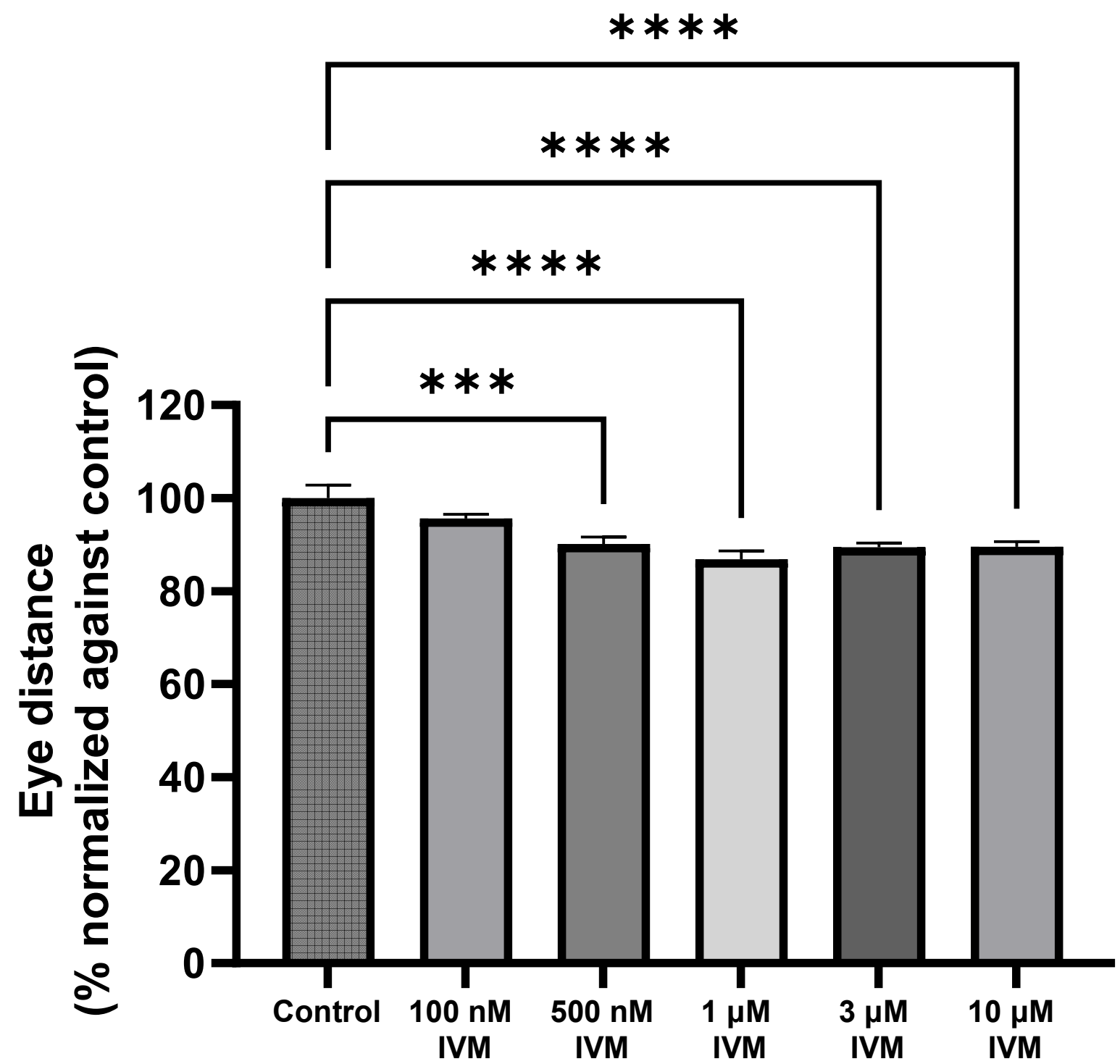

Supplementary figure 2. **Eye distance measurement on increasing concentrations of IVM.** Distance measurement between eyes on treated tadpoles compared to control tadpoles. Length is normalized against control tadpoles. N=7 Ordinary one-way ANOVA test. (*** P<0.001, **** P<0,0001) The error bars of the data correspond to the standard error of the mean (SEM).

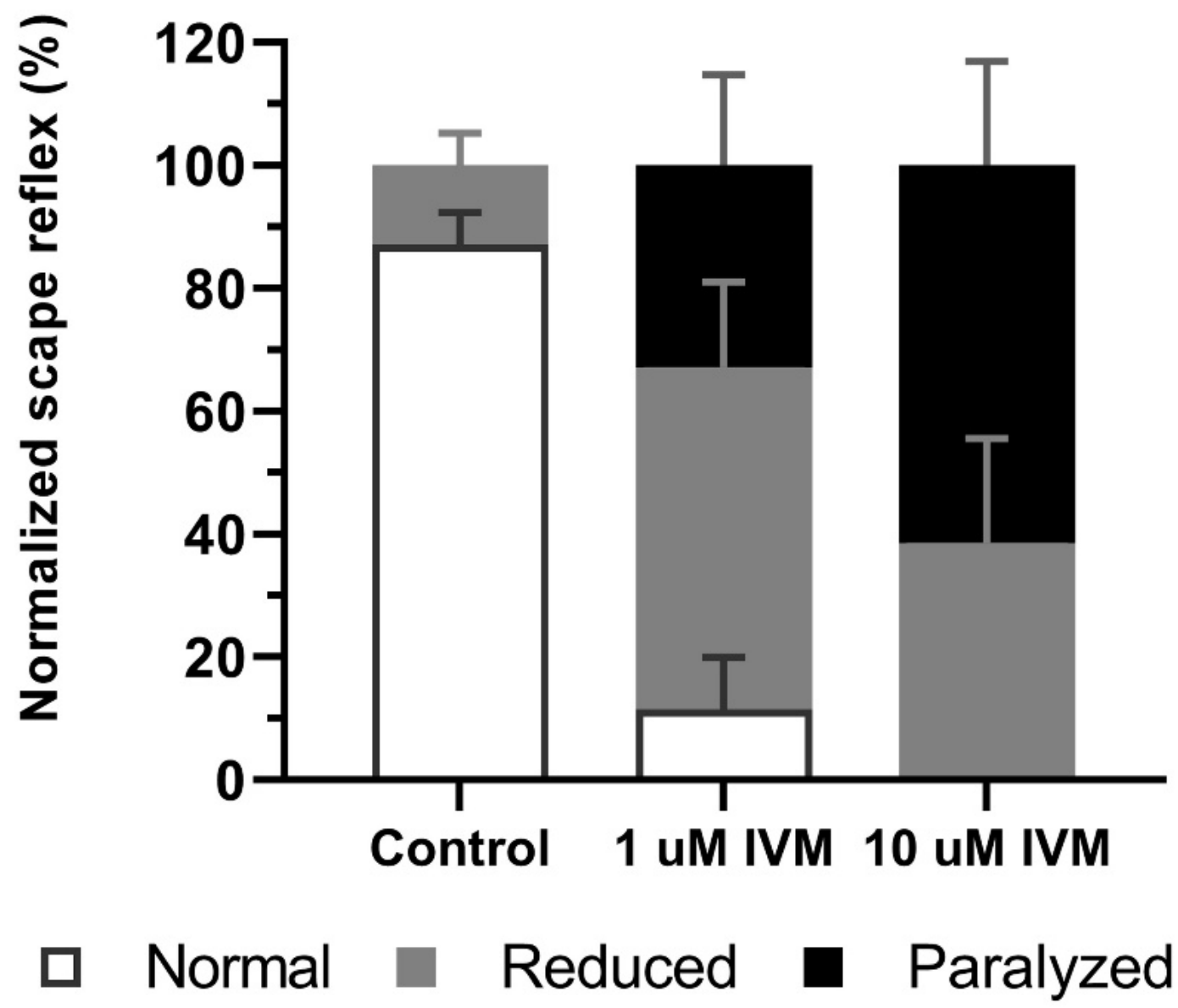

Figure 3. **Effect of IVM on touch-evoked escape reflex of *Xenopus laevis* stage 45 tadpole.** Tadpoles were assigned to three groups depending on their response: Normal (white), reduced (grey) and paralyzed (black). Results are reported as percentage of total batches of embryos analyzed. Number of batches analyzed = 7, 10 embryos per condition. The error bars of the data correspond to the standard error of the mean (SEM). (Spencer et al. 2019)

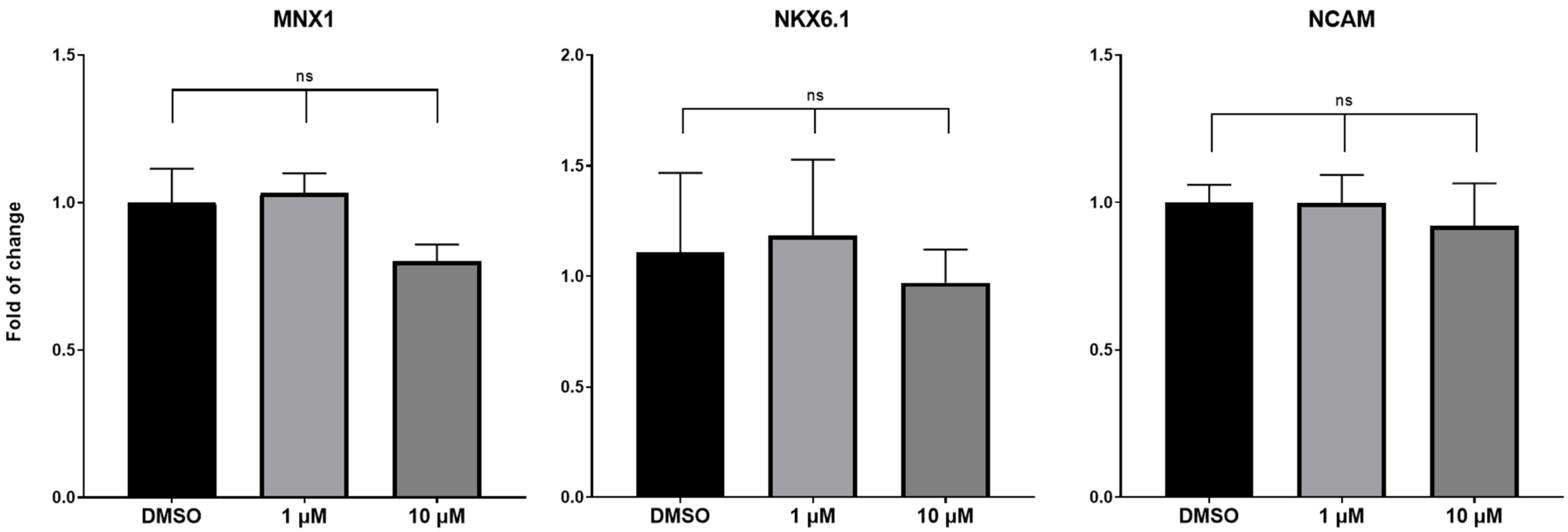

Supplementary figure 4. **Relative expression of motoneurons related transcription factors at tadpole stage 45.** Fold of change of NKX 6.1, NCAM and MNX1 transcripts of IVM treated conditions (1 and 10 µM) compared with control (DMSO) through RT-qPCR assays. N=3. One-way ANOVA, Dunnet's correction, (ns=not significative). The error bars of the data correspond to the standard error of the mean (SEM).

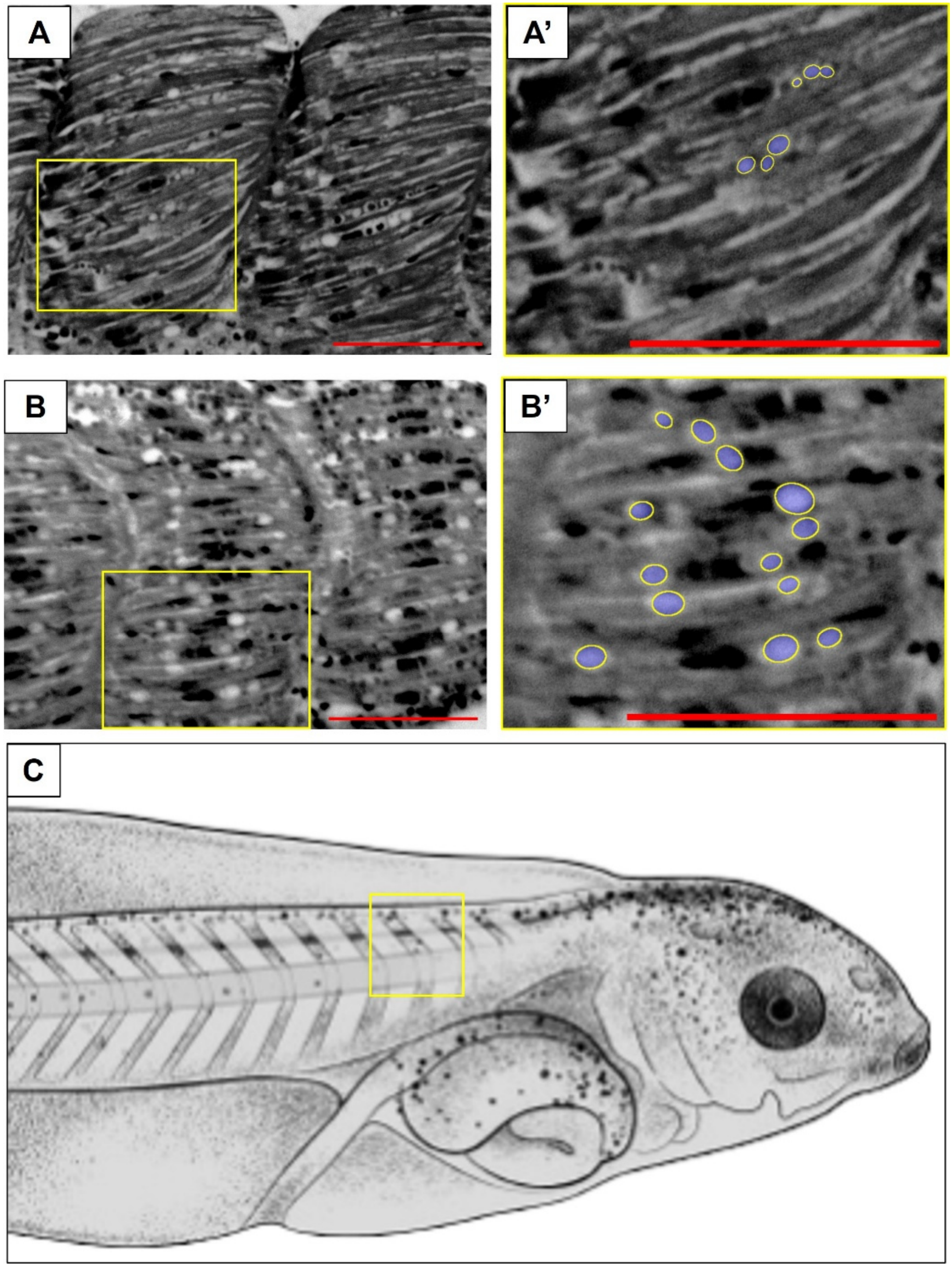

Supplementary figure 5. **Longitudinal hematoxylin & eosin muscle sections of chevron 4-5 of controls and neurula-IVM treatment of stage 45 tadpoles. (A)** Typical pattern of horizontal muscle fibers distribution on control tadpoles. **(A’)** Zoom on vacuolized presented on control section. **(B).** Representative Muscle fibers organization on neurula-IVM treated (10 µM) tadpoles. **(B’)** Zoom of area showing an altered number of muscle fibers vacuolized presented on 10 µM IVM treatment tadpoles. **(C)** Ilustrated tadpole with yellow box indicating the region shown on A, A’, B, B’ scale bar:100 µm. *Xenopus* illustrations © Natalya Zahn (2022).

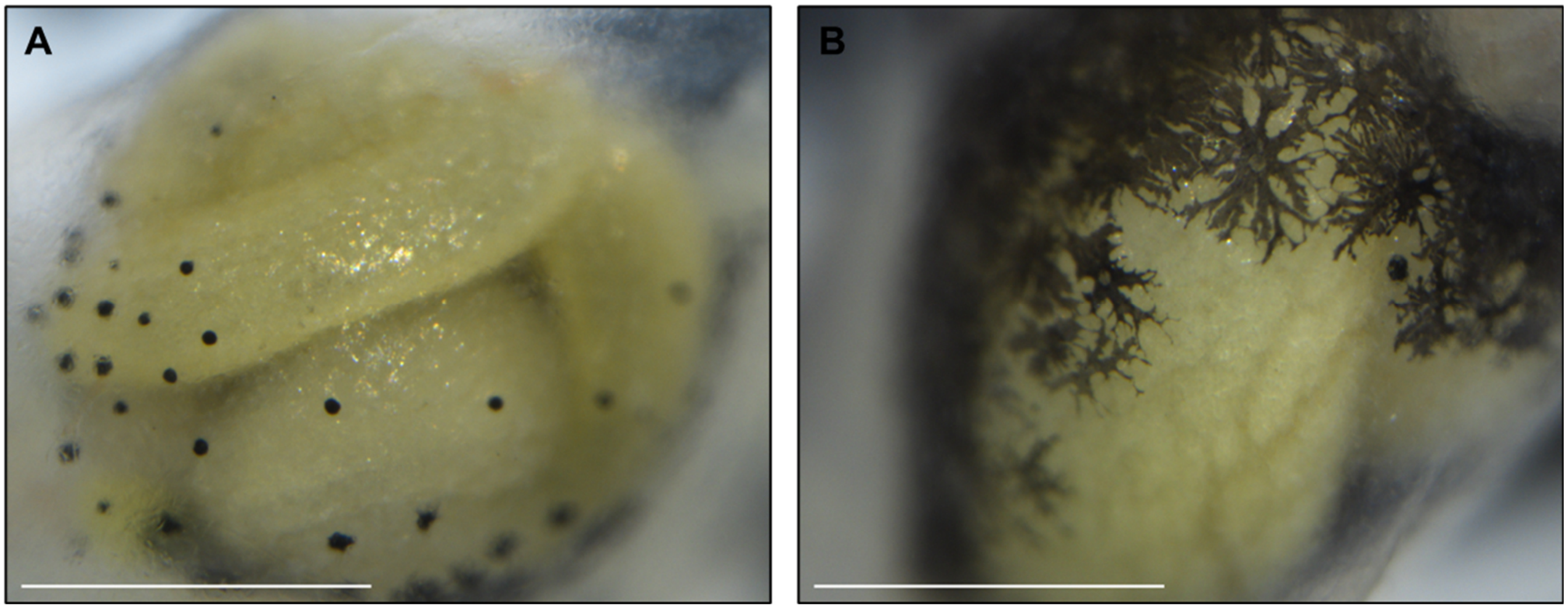

Supplementary figure 6. **Effect of 100 µM ivermectin on digestive system of stage 45 Xenopus tadpoles. (A)** Ventral view of consolidated digestive system and digestive tube in control tadpoles. Typical normal melanocyte phenotype (dark cells) is presented **(B)** Ventral view of alter digestive system on 100 µM IVM neurula-treated tadpoles, in which the digestive tube cannot be identified. Hyper arborized melanocyte phenotype (dark star shape cells) is presented. A, B scale bar: 100 µm.

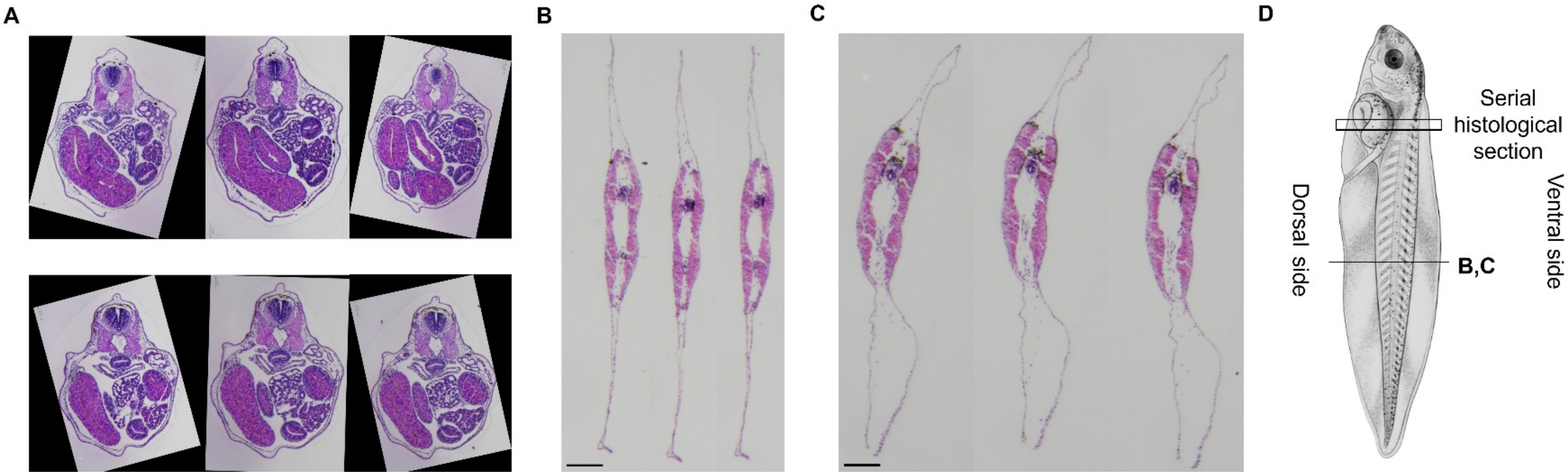

Supplementary Figure 7. **Detailed serial transverse histological sections. (A)** Complete image of serial sections from *Xenopus laevis* tadpoles. The upper panels correspond to the control group. The lower panel corresponds to the IVM-treated tadpoles. Tail sections were also compared: **(B)** shows the control group and **(C)** shows the IVM-treated group. (D) Schematic diagram of a *Xenopus laevis* tadpole showing the sectioning points. A Scale bar: 100 µm. B,C scale bar: 50 µm. *Xenopus* illustrations © Natalya Zahn (2022).

Transcription factors	
Subunit	Sequence (5'-3')
mnx1.L F	TTCTGACTGCACATCCGAGG
mnx1.L R	GGGTGATACTGGCTCAGTGG
ncam1.L F	ACAGATGTCAAGACCCC
ncam1.L R	ATTCCCAATAACACCACCCCT
nkx6-1.L F	CCGGAAGGACTCCCATCTTT
nkx6-1.L R	AACACAGAGCCCTGATTGGG

Supplementary table 1. Primer sequences used in stage 45 tadpole. R=Reverse primer, F=Forward primer, S=Short, L=Long.
